# Effect of Two-Step Sous Vide Cooking and Storage on Microbiological and Oxidative Stability of Chicken Breast

**DOI:** 10.3390/foods12061213

**Published:** 2023-03-13

**Authors:** Endrit Hasani, Gabriella Kiskó, István Dalmadi, Géza Hitka, László Ferenc Friedrich, György Kenesei

**Affiliations:** 1 Department of Livestock Products and Food Preservation Technology, Institute of Food Science and Technology, Hungarian University of Agriculture and Life Sciences, Ménesi út 43-45, 1118 Budapest, Hungary; 2 Department of Food Technology and Biotechnology, Faculty of Agriculture and Veterinary, University of Prishtina, 10000 Prishtina, Kosovo; 3 Department of Food Microbiology, Hygiene, and Safety, Institute of Food Science and Technology, Hungarian University of Agriculture and Life Sciences, Somlói út 14-16, 1118 Budapest, Hungary; 4 Department of Postharvest, Commerce, Supply Chain and Sensory Science, Institute of Food Science and Technology, Hungarian University of Agriculture and Life Sciences, 43-45 D. Ménesi Street, 1118 Budapest, Hungary

**Keywords:** meat, mild heat treatment, shelf life, lipid oxidation, *Enterococcus faecalis*, storage temperature

## Abstract

A two-step sous vide method, which included a low temperature initial stage, was shown to improve texture parameters, increase the solubility of proteins, and decrease the cook loss in chicken breasts. The current work was designed to determine the effect of two-step sous vide and subsequent storage on the microbiological and oxidative stability of chicken breasts. Inoculated chicken breasts were vacuum packaged and cooked at two temperatures, 50 °C and 60 °C, combined in different ratios of the same total cooking time (120 min), and then stored for 21 days at 4 °C, 10 °C, and −20 °C, and compared with the one-step temperature treatment (60 °C for 120 min). One-step sous vide treatment resulted in the total inactivation of *Enterococcus faecalis* NCAIM B. 01312. Meanwhile, the two-step sous vide treatments resulted in a higher than 3 log reduction in *Enterococcus faecalis* NCAIM B. 01312, reaching the target pasteurization performance criterion of sous vide for poultry meat. Lipid oxidation and the odor of all chicken breasts remained acceptable for 21 days of storage at 4 °C and −20 °C. Conversely, all chicken breasts had higher lipid oxidation rates and odor after 21 days of storage at 10 °C. Two-step-sous-vide-treated chicken breasts were found to be microbiologically stable regarding *Enterococcus faecalis* NCAIM B. 01312 and total mesophilic aerobic counts during 21 days of storage at 4 °C and −20 °C, in contrast with those stored at 10 °C. It can be concluded that two-step-sous-vide-cooked chicken breasts had acceptable oxidative and microbiological stability during chilled and frozen storage, similar to one-step sous vide ones. These outcomes highlight that two-step heat treatment can be used as an alternative cooking method to improve the quality properties without compromising the storage life of chicken breasts.

## 1. Introduction

Sous vide mild heat treatment has had growing attention in recent years in the catering sector, households, and among researchers, as it presents a feasible option to obtain higher yields of meat, improve sensorial characteristics such as juiciness and tenderness, and oxidative stability, and enhance the shelf life of meat products [1,2]. The traditional sous vide cooking method employs only one cooking temperature in the range of 55–70 °C for different time durations depending on the type, thickness, and amount of connective tissue present in the meat [3]. Different meat proteins, which have different denaturation temperatures, are responsible for the main quality attributes of cooked meat. In this context, the selection of the proper temperature and time durations in sous vide processing allows the possibility to tailor the denaturation of meat proteins to achieve the desired tenderness and juiciness of cooked meat [4,5]. For example, the endogenous proteolytic enzymes of meat (μ-calpain, μ/m-calpain, and cathepsin B), whose highest activity is between temperatures of 40 to 50 °C, have been shown to extend meat tenderization through desmin degradation [6,7]. μ-Calpain plays an essential role in the tenderization of chicken breast muscle because of its high proteolytic effect, which can be explained by high amounts and a higher activation rate of μ-calpain in breast muscle compared to μ/m-calpain [8]. Furthermore, this study found a correlation between desmin degradation and μ-calpain activity in chicken breast muscles [8]. On the other hand, it has been reported that cathepsins, due to their short proteolytic activity, have little effect on chicken breast tenderization [9]. The main factors affecting the efficiency of proteolytic enzymes (μ-calpain, μ/m-calpain, cathepsins) are pH and ionic strength [10]. As a result, the application of proteolytic enzyme activation temperatures as an initial stage in the sous vide processing can potentially increase chicken breast tenderness. In fact, previous studies demonstrated that a two-step sous vide method that included a temperature of 45–50 °C showed improved texture attributes of meat such as shear force, hardness, gumminess, and chewiness values compared to the traditional one-step sous vide cooking method [11,12,13].

Due to the overlapping temperatures of proteolytic enzyme activation and bacterial growth, special attention is needed to ensure microbial safety of two-step-sous-vide-cooked meat. Currently, there is a lack of data in the literature to predict the growth of bacteria in the range of 42–55 °C [14]. Therefore, it is necessary to test the efficiency of the two-step sous vide treatment using a high thermal-resistant microorganism to optimize the temperature and time combinations in sous vide for pasteurization of the meat product. *Enterococcus* species are the most heat-resistant microorganisms compared to other pathogenic bacteria such as *Escherichia coli* O157: H7, *Salmonella enterica,* and *Listeria monocytogenes* [15,16,17]. However, there has been little research on the presence of Enterococcus species in ready-to-eat foods because most studies have focused on their presence in raw foods. In a recently published study, *Enterococcus faecalis* was found to be the most widespread strain among *Enterococcus* in ready-to-eat meat products with nearly 48.7% followed by *Enterococcus faecium* with 39.7% [18]. Koluman et al. [19] reported that 20% of ready-to-eat chicken was contaminated with Enterococcus spp. Meanwhile, Yuksek et al. [20] reported lower than 2 log CFU/g Enterococcus counts on ready-to-eat chicken donair.

On the other hand, the storage stability of meat products produced with this technique needs to be studied as storage time can have a significant effect on the physical and chemical properties of cooked meat. Hong et al. [21] reported that chilled storage resulted in a loss of moisture and a decrease in water-holding capacity, which may be responsible for the decrease in tenderness in cooked chicken breast. Furthermore, bacteria present on the surface of the meat can multiply, leading to spoilage and off-flavors [22,23]. The overall quality of the meat can also be affected by oxidation, which can cause changes in color, texture, and flavor [24,25]. Archile-Contreras and Purslow [26] reported that the production of reactive oxygen species (ROS) decreases collagen synthesis, thus reducing its solubility, which increases meat toughness. Therefore, the current study was performed to (1) test the efficiency of the two-step sous vide treatment in which 50 °C temperature was included, on the microbiological inactivation of *Enterococcus faecalis* in chicken breast and compare it with the one-step sous vide technique, and (2) examine the storage life of the studied sous-vide-cooked chicken breast. Storage stability was analyzed in a chilled temperature at 4° C, a mild chilled temperature at 10 °C, and a freezing temperature at −20°C. The microbiological effect of sous vide treatment and storage was investigated by the analysis of total mesophilic aerobic counts and *Enterococcus faecalis* counts. On the other hand, the oxidative stability of the cooked chicken breast during storage was investigated using the TBARS method. In addition, the sensory quality of cooked chicken breast was analyzed as olfactory, which presents a pre-indicator of microbiological deterioration and oxidative rancidity [2,27].

## 2. Materials and Methods

### 2.1. Raw Meat Sample Preparation

Fresh *Pectoralis major* muscles (24 h post-mortem) were bought in a local slaughterhouse and transported to the Department of Livestock Products and Food Preservation Technology, Institute of Food Science and Technology, Hungarian University of Agriculture and Life Sciences (Budapest, Hungary), in an ice-filled thermos cool box. *Pectoralis major* muscles represent a suitable raw material for meat research analysis because of their size and homogeneous structure. The samples were deskinned, separated from fat and connective tissue, and cut in uniform weight (129.6 ± 2.4 g) and thickness (2.0 ± 0.3 cm).

#### Preparation of Bacterial Strain and Inoculum

The *Enterococcus faecalis* strain (NCAIM B. 01312) used in the present study was obtained from the National Collection of Agricultural and Industrial Microorganisms (NCAIM, Hungarian University of Agriculture and Life Sciences, Budapest, Hungary). Recovery of the lyophilized culture was performed in Brain Heart Infusion (BHI, Sigma-Aldrich, Munich, Germany) broth and incubated for 24 h at 37 °C, following the procedure described by McAuley et al. [17]. The culture was streaked on the selective *Enterococcus faecalis* agar and incubated for 24 h at 37 °C. Pure cultures were maintained on Tryptic-Soy Agar (TSA, Biokar Diagnostic BK046HA, Sigma-Aldrich) slants at refrigeration conditions (4° C) until use. The first step of preparation of *Enterococcus faecalis* inoculum was the preparation of an overnight culture on the selective Citrate azide tween carbonate (CATC) Agar. After that, a cell suspension of the culture was prepared using MRD diluent and the cell concentration was set to 6–7 log CFU/mL using a McFarland densitometer.

### 2.2. Experimental Design and Sous Vide Treatments

Experiments in the current study were conducted to study the effect of using two temperatures at different time ratios in sous vide processing of chicken breast muscles. Chicken breasts were subjected to vacuum packaging in PA/PE pouches (200 × 250 mm^2^) using a vacuum sealer (Multivac C100, MULTIVAC Sepp Haggenmüller SE & Co. KG, Wolfertschwenden, Germany). Chicken breast samples were cooked at one temperature treatment (T1) and at two temperatures (T2 and T3) by combining a low temperature initial stage and then an end step temperature (Table 1). The study was conducted with three replicates for each sous vide treatment using a two-way completely randomized design.

Heat treatments were conducted using thermostatic water bath (Labor Műszeripari Művek LP507/01). The internal temperature of chicken breasts during treatments was monitored using a needle probe T-type thermocouple, which was placed at the thickest part of the vacuum-packaged chicken breast sample. After treatment, chicken breast samples were cooled down in ice-cold water (1 °C) and were kept at chilled temperature of 2 °C to achieve a lower than 4 °C temperature within 6 h as described in the recommended guidelines of BC Centre for Disease Control [28].

The storage stability of the studied sous vide chicken breasts was examined when stored in chilled temperature at 4 ± 0.5 °C, mild chilled temperature at 10 ± 0.5 °C, and freezing temperature at −20 ± 0.5 °C for up to 21 days. Separate samples were taken for lipid oxidation and odor acceptability analysis and separate samples for microbiological analysis on days 0, 7, 14, and 21 days.

### 2.3. Microbiological Analysis

Microbiological analysis was conducted to check the thermal inactivation of the studied treatments on chicken breast by using *Enterococcus faecalis* as a reference microorganism. Total mesophilic aerobic counts were also analysed after each processing sous vide treatment. *Enterococcus faecalis* and total mesophilic aerobic counts were also tested during the storage of cooked chicken breasts at temperatures of 4° C, 10 °C, and −20 °C for up to 21 days.

#### 2.3.1. Determination of Enterococcus Faecalis

The microbiological challenge test was used to determine the pasteurization level of the chicken breast samples treated with the studied sous vide treatments. *Enterococcus faecalis* was selected as a target microorganism to conduct the thermal inactivation challenge tests.

##### Bacterial Inoculation on Chicken Breast

Approximately 10 g chicken breast sample was inoculated with 0.1 mL *Enterococcus faecalis* B. 01312 bacterial cell suspension with an initial cell count of 6.85 ± 0.5 log CFU/g, as described by Belleti et al. [29]. A negative control sample was also prepared without addition of bacterial cell suspension. The chicken breast samples were then vacuum packaged using a vacuum sealer (Multivac C100, MULTIVAC Sepp Haggenmüller SE & Co. KG, Wolfertschwenden, Germany) and then sous vide treated in a water bath in one of the treatment conditions. The cooked chicken breast was stored at chilled (4 °C), mild chilled (10 °C), or freezing temperature (−20 °C) until the sampling day depending on the experiment.

##### Microbiological Enumeration

After each storage sampling day, each vacuum-packaged, sous-vide-treated chicken breast sample was suspended aseptically with 90 mL Maximum Recovery diluent (MRD, Sigma-Aldrich, Munich, Germany) and homogenized in a stomacher bag for 2 min using a stomacher. After that the samples were 10-fold serially diluted in MRD diluent and were plated on Citrate azide tween carbonate Agar (CATC Agar, Sigma-Aldrich, Munich, Germany) by pour plating of 0.1 mL of sample/dilution and spreading with a sterile glass spreader. The inoculated selective *Enterococcus faecalis* Agar (CATC Agar) plates were then incubated at 37 °C for 24 h and the red obtained colonies were counted using a colony counter. The results were presented as the logarithms of colony-forming units per gram of sample (log CFU/g).

#### 2.3.2. Determination of Total Mesophilic Aerobic Counts

Total mesophilic aerobic counts were determined following the method as described by Jriidi et al. [30]. Ten grams of each chicken breast sample was obtained aseptically, mixed with 90 mL diluent in a stomacher bag, and homogenized for 2 min in a stomacher. Ten-fold serial dilutions were performed using diluent solution and 1 mL was plated onto Plate Count Agar (PCA, Sigma-Aldrich, Munich, Germany). Total mesophilic aerobic counts were determined by incubation of the plates in aerobic conditions at 37 °C for 48 h. The obtained colonies were enumerated by using a colony counter and the results were given in log CFU/g.

### 2.4. Odor Acceptability

During the storage experiment, on each sampling day, the pouches were aseptically open and the samples were evaluated for odor acceptability, as described by other authors [22,31,32]. The samples were scored using a five-point scale: 1 = acceptable, 2 = slightly acceptable, 3 = neutral, 4 = slightly unacceptable, 5 = unacceptable. The sensory panel was a trained professional team, which consisted of 5 members of researchers, teachers, and laboratory assistants of Department of Livestock Products and Food Preservation Technology, Hungarian University of Agriculture and Life Sciences, Budapest, Hungary.

### 2.5. Lipid Oxidation

Lipid oxidation of chicken breast samples was assessed by measuring the thiobarbituric Acid Reactive Substances (TBARS) values as described by Dias et al. [33] with few modifications. TBARS values were expressed as the mean of triplicates for each sample in mg MDA per kilogram of chicken breast [34,35].

### 2.6. Statistical Analysis

The experimental data were analysed using IBM SPSS (Version 27.0, Armnouk, New York, NY, USA, 2020). Data were analysed using the analysis of variance (ANOVA) and General Linear Model (GLM). Kolmogorov–Smirnov test was used to test the normality of residuals (*p* > 0.05). Levene’s test was performed to check the homogeneity of variances (*p* > 0.05). The differences between groups were analysed using Tukey’s post hoc tests if homogeneity of variances was not violated. Meanwhile, if this assumption was violated, then Games–Howell test was performed. The obtained microbiological data were converted to log CFU/g.

## 3. Results

### 3.1. Effect of Sous Vide Treatments on Microbial Inactivation

In the microbiological inactivation experiment of sous-vide-treated chicken breasts, around 6.45 ± 0.08 log CFU/g *Enterococcus faecalis* NCAIM B. 01312 was inoculated in raw samples before treatments. For the pasteurization of food products, a 6 log reduction of the pathogenic bacteria is defined as a pasteurization performance criterion (NACMCF) [36]. Based on our results, the one-step sous vide treatment (T1) of chicken breast samples reduced *Enterococcus faecalis* NCAIM B. 01312 and total mesophilic aerobic counts to non-detectable levels. Similarly, Haghighi et al. [37] reported no total mesophilic counts after sous vide treatments of chicken breast under various temperature (60 to 80 °C) and time (60 to 150 min) combinations.

On the other hand, two-step-sous-vide-treated chicken breasts (T2 and T3) resulted in a higher than 3 log reduction of Enterococcus faecalis NCAIM B. 01312, having 3.01 ± 0.07 and 3.37 ± 0.07 log CFU/g, respectively (Table 1). The thermal inactivation of T2 and T3 treatments can be considered effective for pasteurization taking into account that initial counts of Enterococcus faecalis in raw chicken breast (without inoculation) were around 2.69 ± 0.12 log CFU/g. Similarly, Miranda et al. [38] reported 1–3 log CFU/g Enterococcus counts in raw poultry meat (chicken and turkey). These results showed that the two-step sous vide technique was sufficient to produce safe cooked chicken breasts that had higher quality attributes compared to those of one-step sous vide [11].

### 3.2. Oxidative Storage Stability

**Figure 1:**
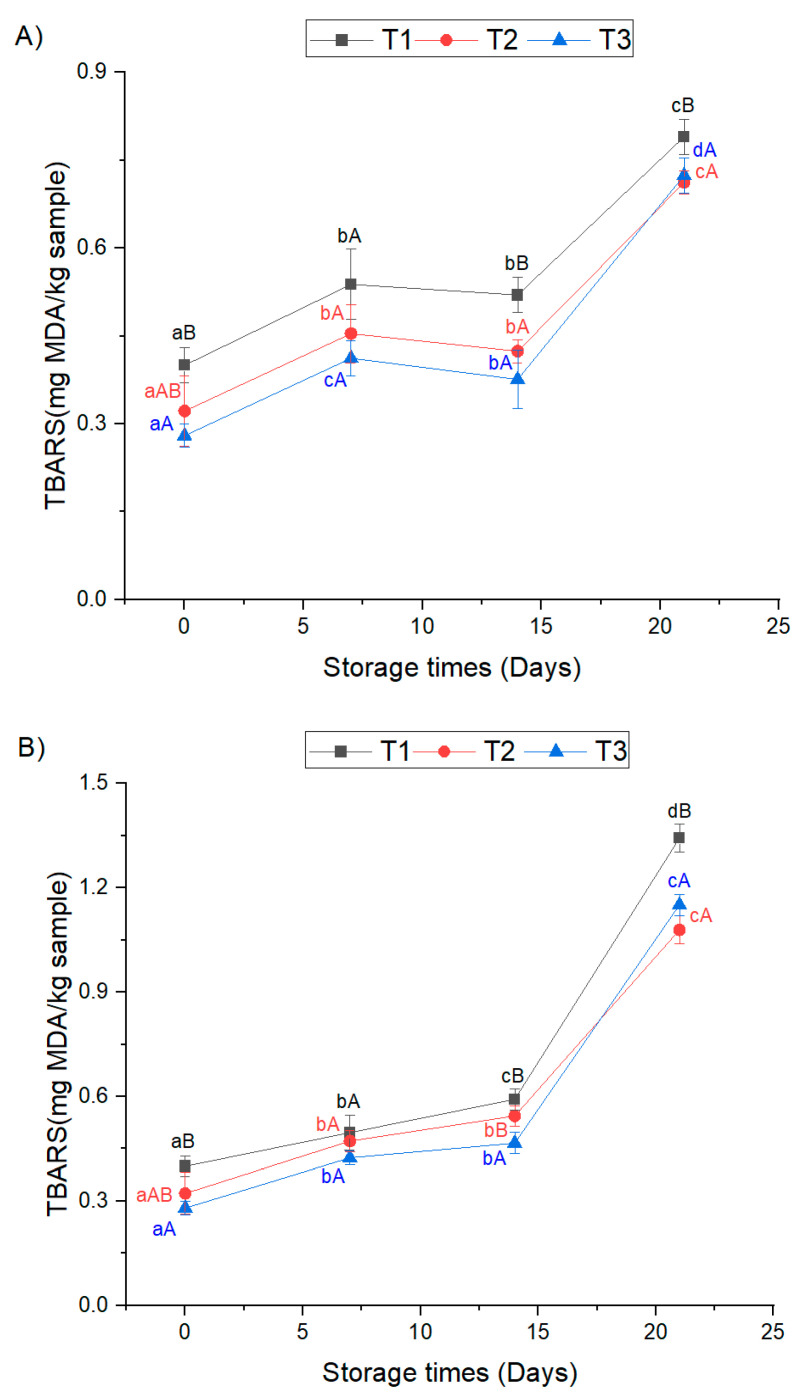

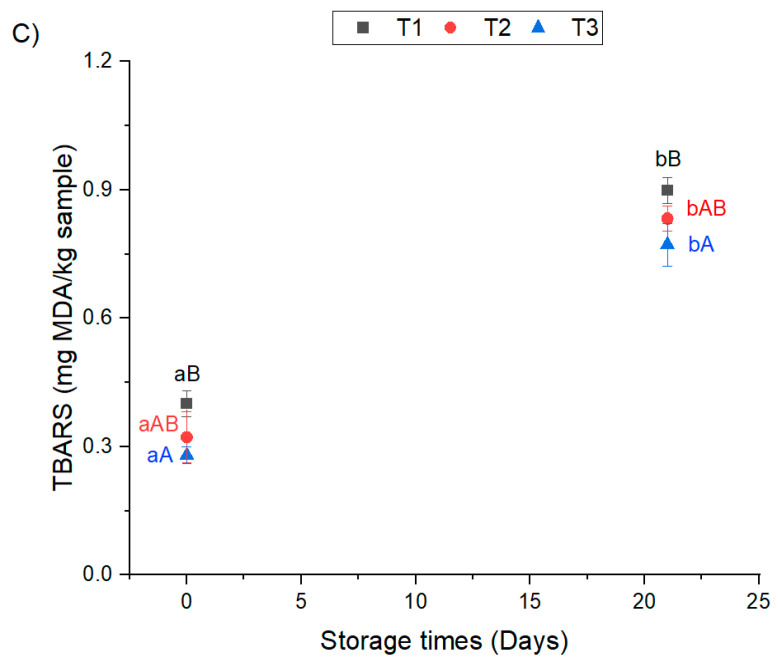
TBARS values (mg MDA/kg sample) of sous-vide-treated chicken breasts stored at 4 °C (**A**), 10 °C (**B**), and −20 °C (**C**) for 21 days. T1: 60 °C/120 min; T2: 50 °C/40 min + 60 °C/80 min; T3: 50 °C/60 min + 60 °C/60 min. a,b,c means with different letters are significantly different for storage days. A, B, C means with different letters are significantly different for treatment type (*p* < 0.05).

TBARS values measure the amount of secondary lipid oxidation products, specifically malonaldehyde, which is known to have a major effect on the aroma deterioration of meat products during storage. In the current study, TBARS values of all the studied sous-vide-treated chicken breasts were significantly increased after 7 days of chilled storage at 4 °C (*p* < 0.05). Furthermore, a significant increase in TBARS values was observed between 14 and 21 days of chilled storage in all sous vide treatments (*p* < 0.05) (Figure 1a). According to the study of Hong et al. [21], the TBARS values of cooked chicken breast were significantly increased after 10 days of chilled storage at 4 °C. Conversely, Akoğlu et al. [27] reported that the TBARS values of sous-vide-treated turkey cutlets were significantly increased after 21 days of storage at 4 °C.

On the other hand, the TBARS values of all the studied sous vide chicken breasts were significantly increased after 7 days of mild chilled storage at 10 °C (*p* < 0.05). Akoğlu et al. [27] reported a significant increase in lipid oxidation of sous-vide-cooked turkey cutlets after 12 days of storage at 12 °C.

The highest values of TBARS were noticed in one-step-sous-vide-treated chicken breasts (T1 treatment) after 21 days of mild chilled storage with 1.34 ± 0.04 mg MDA/kg of the sample. Meanwhile, the lowest TBARS value, 0.28 ± 0.02 mg MDA/kg, was observed for the two-step sous vide treatment (T3) at day 0. It has been reported that the threshold level of TBARS for consumers to detect oxidative rancidity in meat is higher than 1 mg malonaldehyde per kilogram of a sample [27]. Based on our study results, this threshold level was exceeded only in sous-vide-treated chicken breast after mild chilled storage at 10 °C for 21 days. It can be emphasized that chilled and frozen storage led to greater stability in lipid oxidation compared with mild chilled storage. Furthermore, the low lipid oxidation rates observed in our study can be explained by the low levels of fat in chicken breast muscles, the heat treatment parameters, and the effect of vacuum packaging on the prevention of lipid oxidation.

TBARS values of chicken breasts cooked with the T1 treatment were significantly higher during each sampling day of mild chilled storage at 10 °C (*p* < 0.05). In general, chicken breasts cooked with the T3 treatment had significantly lower TBARS values than those cooked with the T1 treatment at all sampling days (*p* < 0.05) except at 7 days of chilled and mild chilled storage. At the end of both the chilled and mild chilled storage, the TBARS values of two-step sous vide chicken breasts were significantly lower than the one-step-sous-vide-treated ones (*p* < 0.05). This can be explained by the low oxidation rates of the chicken breast after being cooked with low temperature treatments at day 0 compared to the traditional sous vide one [11].

According to the results presented in Figure 1c, the TBARS values of all the sous-vide-treated chicken breasts were significantly increased during 21 days of frozen storage at −20 °C (*p* < 0.05). This finding suggests that freezing is insufficient to prevent lipid oxidation in meat. Water that does not freeze during frozen storage is available for chemical reactions such as lipid and protein oxidation. The accumulation of lipid oxidation products in muscle is thought to occur as a result of ice crystal damage to cell membranes and the release of pro-oxidants such as haem iron, which may cause deterioration of color, flavor, and pigment oxidation in meat products [39,40]. On the other hand, a significant difference was observed in the TBARS values between chicken breasts cooked with the T3 treatment and those cooked with the T1 treatment before and after frozen storage (*p* < 0.05). Apparently, the increase in oxidation rates was similar during frozen storage for the two types of treatments of sous vide chicken breasts. The high oxidative storage stability of the two-step sous vide chicken breasts compared to one-step sous vide ones is an indicator of the higher quality and shelf life of this product.

### 3.3. Odor Acceptability

**Figure 2:**
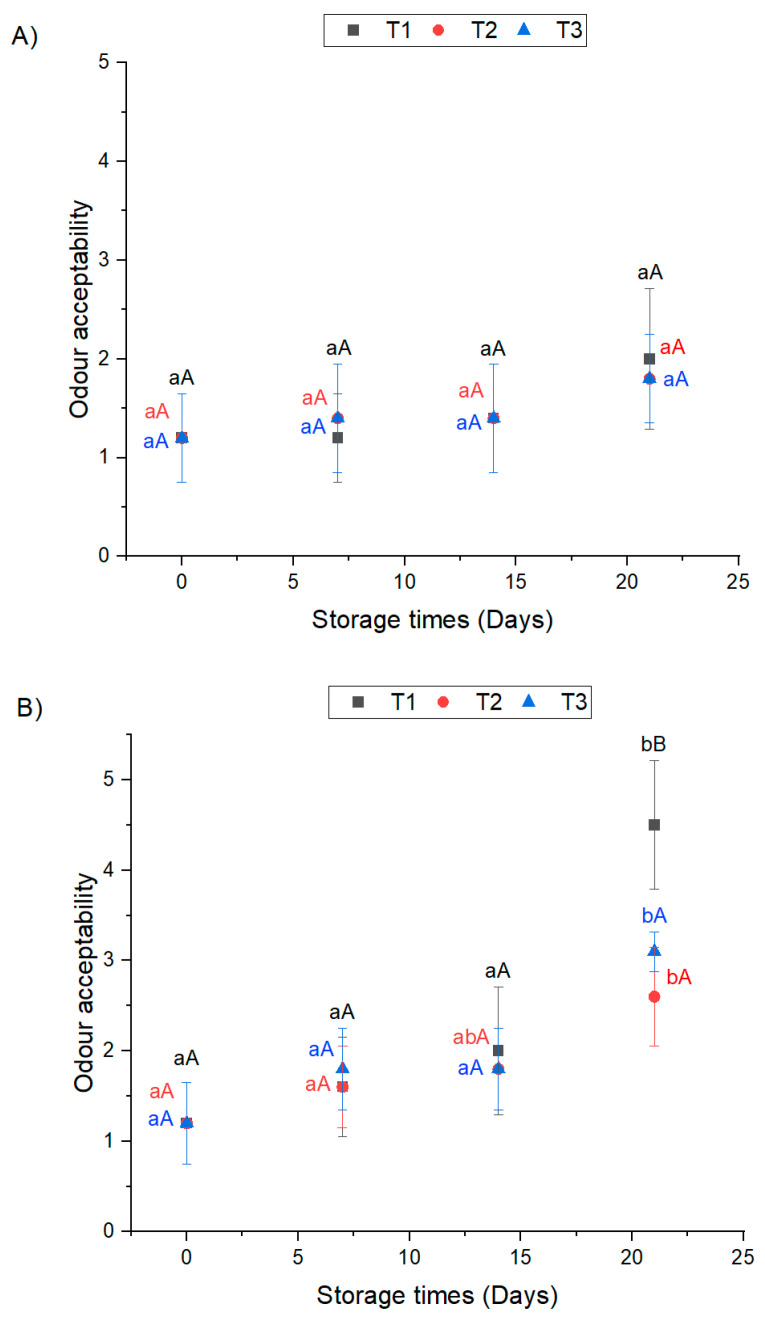

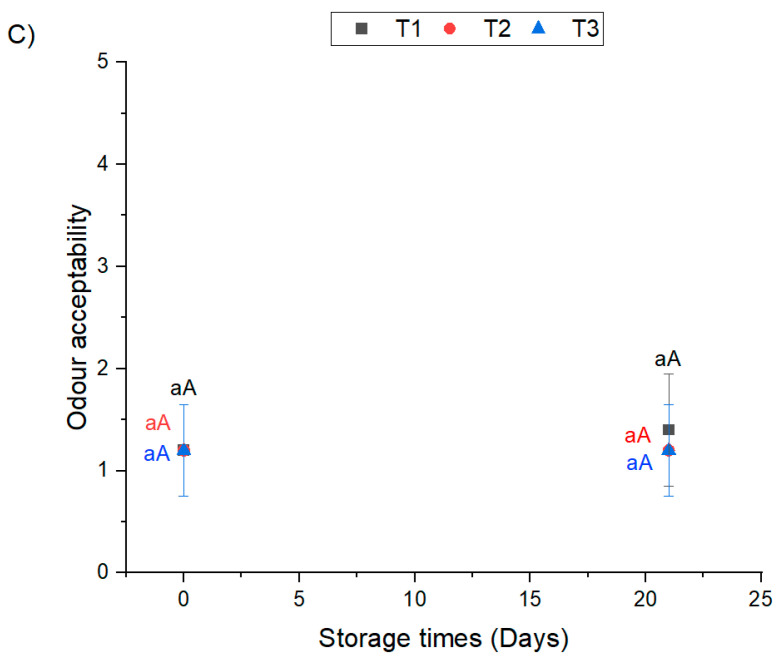
Odor acceptability scores for sous-vide-treated chicken breasts stored at 4 °C (**A**), 10 °C (**B**), and −20 °C (**C**) for 21 days. T1: 60 °C/120 min; T2: 50 °C/40 min + 60 °C/80 min; T3: 50 °C/60 min + 60 °C/60 min. The odor acceptability was evaluated using five-point scale: 1 = acceptable, 2 = slightly acceptable, 3 = neither acceptable nor unacceptable, 4 = slightly unacceptable, 5 = unacceptable. a,b means with different letters are significantly different for storage days. A,B means with different letters are significantly different for treatment type (*p* < 0.05).

Based on the results presented in Figure 2c, the odor of all sous-vide-treated chicken breasts remained acceptable during 21 days of frozen storage at −20 °C with scores lower than 2. Similarly, sous-vide-cooked chicken breasts had acceptable odor during chilled storage at 4 °C except for the T1 treatment, which resulted in it being slightly acceptable after 21 days with a score equal to 2 (Figure 2a).

On the other hand, the odor of sous-vide-treated chicken breasts resulted in being acceptable during 14 days of mild chilled storage at 10 °C except for the one-step sous vide treatment. After 21 days of mild chilled storage, the two-step-sous-vide-treated chicken breasts, T2, had a slightly acceptable odor with acceptance scores of 2.6. Meanwhile, the chicken breasts cooked with treatments T3 and T1 had the highest acceptance scores (3.1 and 4.5, respectively) after 21 days of mild chilled storage at 10 °C. The unacceptable odor of the one-step-sous-vide-treated chicken breasts (T1) can be associated with the development of secondary lipid oxidation products during mild chilled storage, which were over the sensorial threshold limit of 1 mg MDA/kg sample [27] (Figure b). Furthermore, the sensory quality of cooked meat can be a pre-indicator of microbiological spoilage during storage [2].

### 3.4. Microbiological Storage Stability

According to the results presented in Table 2, *Enterococcus faecalis* NCAIM B. 01312 counts did not significantly change during 21 days of chilled storage of the sous-vide-treated chicken breasts (*p* < 0.05). Similarly, Ingham and Tautorus [41] reported no significant change in *Enterococcus faecalis* counts in cooked turkey meat for 15 days storage at 3 ± 1 °C. The highest *Enterococcus faecalis* NCAIM B. 01312 counts were observed after 21 days of chilled storage in T3-sous-vide-cooked chicken breast with 3.4 ± 0.02 log CFU/g, which if it is subtracted from the inoculated counts before heat treatments give a higher than 3 log reduction of *Enterococcus faecalis* NCAIM B. 01312. This shows that the sous-vide-treated chicken breasts were microbiologically stable during 21 days of chilled storage regarding *Enterococcus faecalis* NCAIM B. 01312.

On the other hand, the total mesophilic aerobic counts were significantly increased in the T2 and T3 treatments after 21 days and 14 days of chilled storage, respectively (*p* < 0.05). However, none of the studied sous-vide-treated chicken breasts exceeded the 5 log CFU/g limit during 21 days of chilled storage (Table 3). The chicken breasts cooked with the T2 treatment had significantly lower total mesophilic aerobic counts and Enterococcus faecalis NCAIM B. 01312 compared to chicken breasts cooked with the T3 treatment in all sampling days of chilled storage (*p* < 0.05). This result shows that the T2-sous-vide-treated chicken breasts had the highest microbiological stability during chilled storage at 4 °C of the two-step sous vide treatments.

*Enterococcus faecalis* NCAIM B. 01312 counts of the sous-vide-cooked chicken breasts had an increasing trend during 21 days of mild chilled storage at 10 °C (Table 4). A significant increase in *Enterococcus faecalis* NCAIM B. 01312 counts in the T2-sous-vide-treated chicken breasts was observed only after 7 days of mild chilled storage and after 14 days in samples cooked with the T3 treatment (*p* < 0.05). At the end of the mild chilled storage of the sous-vide-treated chicken breasts, *Enterococcus faecalis* NCAIM B. 01312 counts were increased to 6.37–6.64 log CFU/g, similar to the initial counts inoculated before treatments. This result is expected as *Enterococcus faecalis* has been reported to grow at temperatures as low as 10 °C [42].

On the other hand, total mesophilic aerobic counts in chicken breasts cooked with the T2 and T3 treatments exceeded the limit criteria of 5 log CFU/g after 14 days of mild chilled storage at 10 °C. Total mesophilic counts were significantly increased after 14 days of mild chilled storage of the two-step-sous-vide-cooked chicken breast (*p* < 0.05) (Table 4). Of the two-step sous vide treatments, the chicken breasts treated with treatment T2 had significantly lower *Enterococcus faecalis* NCAIM B. 01312 and total mesophilic aerobic counts compared to the T3 treatment during 21 days of mild chilled storage, with the exception of day 7 (*p* < 0.05).

*Enterococcus faecalis* NCAIM B. 01312 in sous-vide-cooked chicken breast was significantly decreased during 21 days of frozen storage at −20 °C (*p* < 0.05) (Table 5). Similarly, total mesophilic aerobic counts after 21 days of frozen storage were significantly decreased compared to initial counts before storage, thus not exceeding the limit criteria of 5 log CFU/g. Mohammed et al. [43] also reported a significant decrease in total mesophilic aerobic counts in minced chicken meat during frozen storage at −20 °C. Sous-vide-cooked chicken breasts (T2) had significantly lower *Enterococcus faecalis* NCAIM B. 01312 and total mesophilic aerobic counts compared to T3-sous-vide-cooked chicken breasts before and after frozen storage (*p* < 0.05).

## 4. Conclusions

The pasteurization level of both one- and two-step sous vide treatments resulted in being safe based on thermal inactivation of the heat-resistant microorganism *Enterococcus faecalis* NCAIM B. 01312. The one-step sous vide treatment performed at 60 °C for 120 min successfully inactivated *Enterococcus faecalis* NCAIM B. 01312 counts in chicken breast. Meanwhile, chicken breasts cooked for 120 min at two temperatures, 50 °C and 60 °C, achieved the target pasteurization performance criterion of a 3 log reduction in *Enterococcus faecalis* NCAIM B. 01312 in chicken breast.

Regarding oxidative storage stability, all sous-vide-treated chicken breasts had TBARS values within the sensorial threshold limit during 21 days at 4 °C and at −20 °C. Conversely, the TBARS values of all sous-vide-cooked chicken breasts exceeded the sensorial threshold limit after 21 days at 10 °C, which is also supported by high odor acceptability scores. On the microbiology aspect, chicken breasts cooked in two temperatures resulted as being within the criterion limits for *Enterococcus faecalis* NCAIM B. 01312 and total mesophilic aerobic counts only when stored at 4 °C and at −20 °C. The outcomes of the present study along with those reported in the previous study (Hasani et al. [11]) suggest that two-step sous vide can be used as an alternative cooking method to improve the quality properties of chicken breasts and is sufficient to reduce heat-resistant microorganisms at appropriate levels. Furthermore, the storage stability of two-step sous vide chicken breasts is not compromised if stored at a temperature lower than 4 °C, similar to the one-step sous vide ones.

## Figures and Tables

**Table 1:** Sous vide cooking treatment groups.

Group	Time at Temperature of 50 °C (Min)	Time at Temperature of 60 °C (Min)	Treatment Time Ratio 50 °C/60 °C	Total Treatment Time (Min)
T1	0	120	0:1	120
T2	40	80	1:2	120
T3	60	60	1:1	120

**Table 2:** Means ± standard deviations of total mesophilic aerobic counts and *Enterococcus faecalis* NCAIM B. 01312 (log CFU/g) recovered from raw and cooked chicken breasts.

Microorganism	Raw Sample	Treatments			*p*-Value
		T1	T2	T3	
Total mesophilic aerobic counts (log CFU/g)	5.72 ± 0.5	ND	3.77 ± 0.08 ^a^	4.03 ± 0.04 ^b^	0.06
*Enterococcus faecalis* NCAIM B. 01312 (log CFU/g)	2.69 ± 0.12	ND	3.01 ± 0.07 ^a^	3.37 ± 0.07 ^b^	0.02

ND—not detected. T1: 60 °C/120 min; T2: 50 °C/40 min + 60 °C/80 min; T3: 50 °C/60 min + 60 °C/60 min. ^a,b^ means with different letters in the same row are significantly different for treatment type (*p* < 0.05).

**Table 3:** Means ± standard deviations of total mesophilic aerobic counts and *Enterococcus faecalis* NCAIM B. 01312 (log CFU/g) of sous-vide-cooked chicken breast during storage at 4 °C.

Microorganism	Storage Days	Treatments		
		T1	T2	T3
Total mesophilic aerobic counts (log CFU/g)	0	ND	3.77 ± 0.08 ^aA^	4.03 ± 0.04 ^aB^
	7	ND	3.8 ± 0.03 ^aA^	4.12 ± 0.05 ^abB^
	14	ND	3.81 ±0.04 ^aA^	4.19 ± 0.08 ^bcB^
	21	ND	3.95 ±0.03 ^bA^	4.31 ± 0.03 ^cB^
	*p*-value	-	0.008	0.001
*Enterococcus faecalis* NCAIM B. 01312 (log CFU/g)	0	ND	3.01 ± 0.07 ^aA^	3.37 ± 0.07 ^aB^
	7	ND	3.07 ± 0.04 ^aA^	3.40 ± 0.02 ^aB^
	14	ND	3.11 ± 0.02 ^aA^	3.39 ± 0.03 ^aB^
	21	ND	3.09 ± 0.02 ^aA^	3.40 ± 0.02 ^aB^
	*p*-value	-	0.07	0.63

ND—not detected. T1: 60 °C/120 min; T2: 50 °C/40 min + 60 °C/80 min; T3: 50 °C/60 min + 60 °C/60 min. ^a,b,c^ means with different letters in the same column are significantly different for storage days (*p* < 0.05). ^A,B^ means with different letters in the same row are significantly different for treatment type (*p* < 0.05).

**Table 4:** Means ± standard deviations of total mesophilic aerobic counts and *Enterococcus faecalis* NCAIM B. 01312 (log CFU/g) of sous-vide-cooked chicken breast during storage at 10 °C.

Microorganism	Storage Days	Treatments		
		T1	T2	T3
Total mesophilic aerobic counts (log CFU/g)	0	ND	3.77 ± 0.08 ^Aa^	4.03 ± 0.04 ^aB^
	7	ND	3.89 ± 0.04 ^aA^	4.0 ± 0.1 ^aA^
	14	ND	5.02 ± 0.04 ^bA^	5.29 ± 0.03 ^bB^
	21	ND	6.64 ± 0.04 ^cA^	6.91 ± 0.04 ^cB^
	*p*-value	-	<0.001	<0.001
*Enterococcus faecalis* NCAIM B. 01312 (log CFU/g)	0	ND	3.01 ± 0.07 ^aA^	3.37 ± 0.07 ^aB^
	7	ND	3.24 ± 0.06 ^bA^	3.47 ± 0.03 ^aB^
	14	ND	4.22 ± 0.09 ^cA^	5.08 ± 0.05 ^bB^
	21	ND	6.37 ± 0.03 ^dA^	6.64 ± 0.04 ^cB^
	*p*-value	-	<0.001	<0.001

ND—not detected. T1: 60 °C/120 min; T2: 50 °C/40 min + 60 °C/80 min; T3: 50 °C/60 min + 60 °C/60 min. ^a,b,c,d^ means with different letters in the same column are significantly different for storage days (*p* < 0.001). ^A,B^ means with different letters in the same row are significantly different for treatment type (*p* < 0.05).

**Table 5:** Means ± standard deviations of total mesophilic aerobic counts and *Enterococcus faecalis* NCAIM B. 01312 (log CFU/g) of sous-vide-cooked chicken breast during storage at −20 °C.

Microorganism	Storage Days	Treatments		
		T1	T2	T3
Total mesophilic aerobic counts (log CFU/g)	0	ND	3.77 ± 0.08 ^bA^	4.03 ± 0.04 ^bB^
	21	ND	3.1 ± 0.05 ^aA^	3.35 ± 0.06 ^aB^
	*p*-value	-	<0.001	<0.001
*Enterococcus faecalis* NCAIM B. 01312 (log CFU/g)	0	ND	3.01 ± 0.07 ^bA^	3.37 ± 0.07 ^bB^
	21	ND	2.05 ± 0.05 ^aA^	2.91 ± 0.05 ^aB^
	*p*-value	-	<0.001	<0.001

ND—not detected. T1: 60 °C/120 min; T2: 50 °C/40 min + 60 °C/80 min; T3: 50 °C/60 min + 60 °C/60 min. ^a,b^ means with different letters in the same column are significantly different for storage days (*p* < 0.001). ^A,B^ means with different letters in the same row are significantly different for treatment type (*p* < 0.05).

## Data Availability

Data is contained within the article.
